# Clinicopathologic and mutational profiles of primary breast diffuse large B cell lymphoma in a male patient: case report and literature review

**DOI:** 10.1186/s12957-023-03234-z

**Published:** 2023-10-26

**Authors:** Fengbo Huang, Yachao Ruan, Xiaojuan He, Hui Lian, Jinhua Yang

**Affiliations:** 1 Department of Pathology, The Second Affiliated Hospital of Zhejiang University School of Medicine, Hangzhou, China; 2 Key Laboratory of Tumor Microenvironment and Immune Therapy of Zhejiang Province, Hangzhou, China; 3 Department of Radiology, The First Affiliated Hospital of Zhejiang University School of Medicine, Hangzhou, China; 4 Linping Campus, The Second Affiliated Hospital of Zhejiang University School of Medicine, Hangzhou, China; 5 Department of Hematology, The First Affiliated Hospital of Zhejiang Chinese Medical University (Zhejiang Provincial Hospital of Chinese Medicine), Hangzhou, China

**Keywords:** Male, Primary breast diffuse large B cell lymphoma, Clinicopathological features, Genomic mutational profiles

## Abstract

**Introduction:**

Primary breast lymphoma (PBL) is rare, and most cases occur in female patients, with few reported cases in male patients. The clinical presentation is similar to that of breast cancer, but the condition needs to be well understood, as treatment options and clinical course vary. Hence, we provide a relatively rare case of primary breast diffuse large B cell lymphoma (PB-DLBCL) in a male, including its complete clinicopathological features, radiological findings, genomic mutational profiles, and clinical course.

**Case presentation:**

A 45-year-old male presented with a lump in his right breast for 1 week and was pathologically diagnosed with breast malignancy after a breast puncture biopsy at the local hospital. He came to our hospital for further treatment and underwent breast ultrasound and systemic positron emission tomography/computed tomography (PET/CT) imaging, followed by right mastectomy and sentinel lymph node biopsy. Histomorphology showed diffuse hyperplasia of tumor cells with clear boundaries and surrounding normal breast ducts. The adhesion of tumor cells was poor with obvious atypia. Immunohistochemical results showed that the tumor cells were positive for CD20, Bcl6, and MUM-1 but negative for CK (AE1/AE3), ER, PR, CD3, and CD10. Forty percent of the tumor cells were positive for c-Myc, and 80% of tumor cells were positive for Bcl2. The Ki-67 proliferation index was up to 80%. The tumor cells were negative for MYC and BCL2 rearrangements but positive for BCL6 rearrangement by fluorescent in situ hybridization. No abnormality was found in the pathological examination of bone marrow aspiration. Therefore, the male was diagnosed with PB-DLBCL, nongerminal center (non-GCB) phenotype, dual-expression type. The sample were sequenced by a target panel of 121 genes related to lymphoma. Next-generation sequencing revealed six tumor-specific mutated genes (IGH/BCL6, TNFAIP3, PRDM1, CREBBP, DTX1, and FOXO1). The patient was given six cycles of orelabrutinib plus R-CHOP chemotherapy and two cycles of intrathecal injection of cytarabine. The last follow-up was on April 13, 2023 (17 months). No recurrence or metastasis was found in laboratory and imaging examinations.

**Conclusion:**

We reported a relatively rare PB-DLBCL in a male, non-GBC phenotype, dual-expression type. It is worth mentioning that this case had IgH/BCL6 fusion, nonsense mutations in TNFAIP3, frameshift mutations in PRDM1, and missense mutations in CREBBP, DTX1, and FOXO1. To the best of our knowledge, this case is the first report of genomic mutational profiles of PB-DLBCL in males.

**Supplementary Information:**

The online version contains supplementary material available at 10.1186/s12957-023-03234-z.

## Introduction

PBL is a rare neoplasm of the breast that is defined as lymphoma limited to one or both mammary glands with or without regional lymph node metastasis and without a history of lymphoma. It accounts for < 0.5% of all breast malignancies and approximately 2% of all extranodal lymphomas [1, 2]. Most cases occur in middle-aged to elderly women. In the PubMed database, we searched 27 cases of male PBL in the English literature, most of which were case reports [1–23]. Previous reports on male PBL have focused mainly on the clinical and pathological features of the tumor. Reports describing the molecular features of male PBL are scarce. Only Shaymaa Elgaafary et al. [17] reported a case of male breast high-grade B cell lymphoma with MYC and BCL2 rearrangement, and genome-wide chromosomal imbalance mapping revealed a complex pattern of aberrations, including copy-number gains in chromosomes 3q and 18 and focal homozygous loss in 9p21.3, resembling typical changes of lymphomas affecting “immune-privileged” sites.

However, as far as we know, there are no reports in the English literature on the genomic mutational profiles of male PB-DLBCL in the PubMed database. Hence, we reported a rare male PB-DLBCL, the non-GBC type, dual-expression type, and we had a comprehensive description of it, including its complete clinical course, imaging findings, clinicopathological features, and genomic mutational profiles.

## Case presentation

### Clinical history

A 45-year-old man with a right breast mass for 1 week and breast puncture biopsy of pathological diagnosis was breast malignancy in the local hospital. Subsequently, he was admitted to the breast surgery department of our hospital. The patient had no complaints of fever, weight loss, or night sweats and felt no itching, nipple depression or overflow or other discomfort. Blood testing revealed a lactate dehydrogenase level of 289 U/L. Other laboratory tests and tumor markers showed no abnormalities. There was no special medical history.

### Imaging examinations

**Fig. 1:**
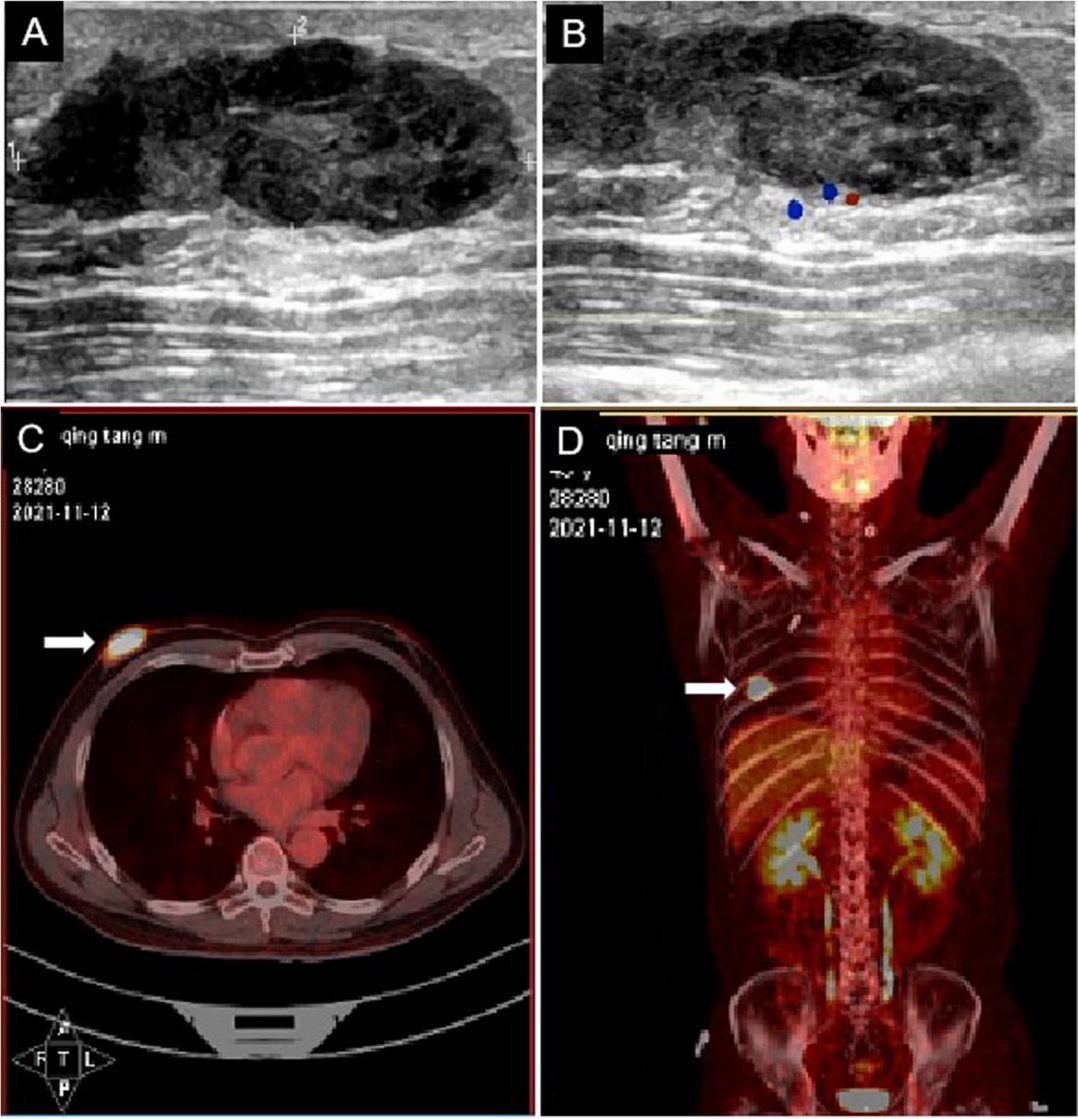
Imaging of primary breast diffuse large B cell lymphoma in a male patient. B-ultrasound of the breast revealed a low-echo mass behind the right nipple with clear boundaries, irregular shape, and uneven internal echo (**A**). Color Doppler flow imaging (CDFI) showed a blood flow signal around the mass (**B**, star). Systemic positron emission tomography/computed tomography (PET/CT) showed abnormally elevated dextrolactose metabolism (**C** and **D**, arrow), which was considered a malignant lesion

Ultrasound showed a hypoechoic mass behind the right nipple, approximately 3.53 cm by 1.07 cm in size, with clear borders, irregular morphology, and uneven internal echogenicity (Fig. 1A). Color Doppler flow imaging showed a peripheral blood flow signal (Fig. 1B), and category 6 was evaluated by the Breast Imaging-Reporting and Data System (BI-RADS) classification method. There was no obviously enlarged lymph nodes were observed in the bilateral axillae. The results of systemic positron emission tomography/computed tomography (PET/CT) imaging showed abnormally elevated foci of right breast glucose metabolism (Fig. 1C, D), and the standardized uptake value (SUV) max was 21.74, indicating malignant lesions.

### Surgical findings and pathological examination results

**Fig. 2:**
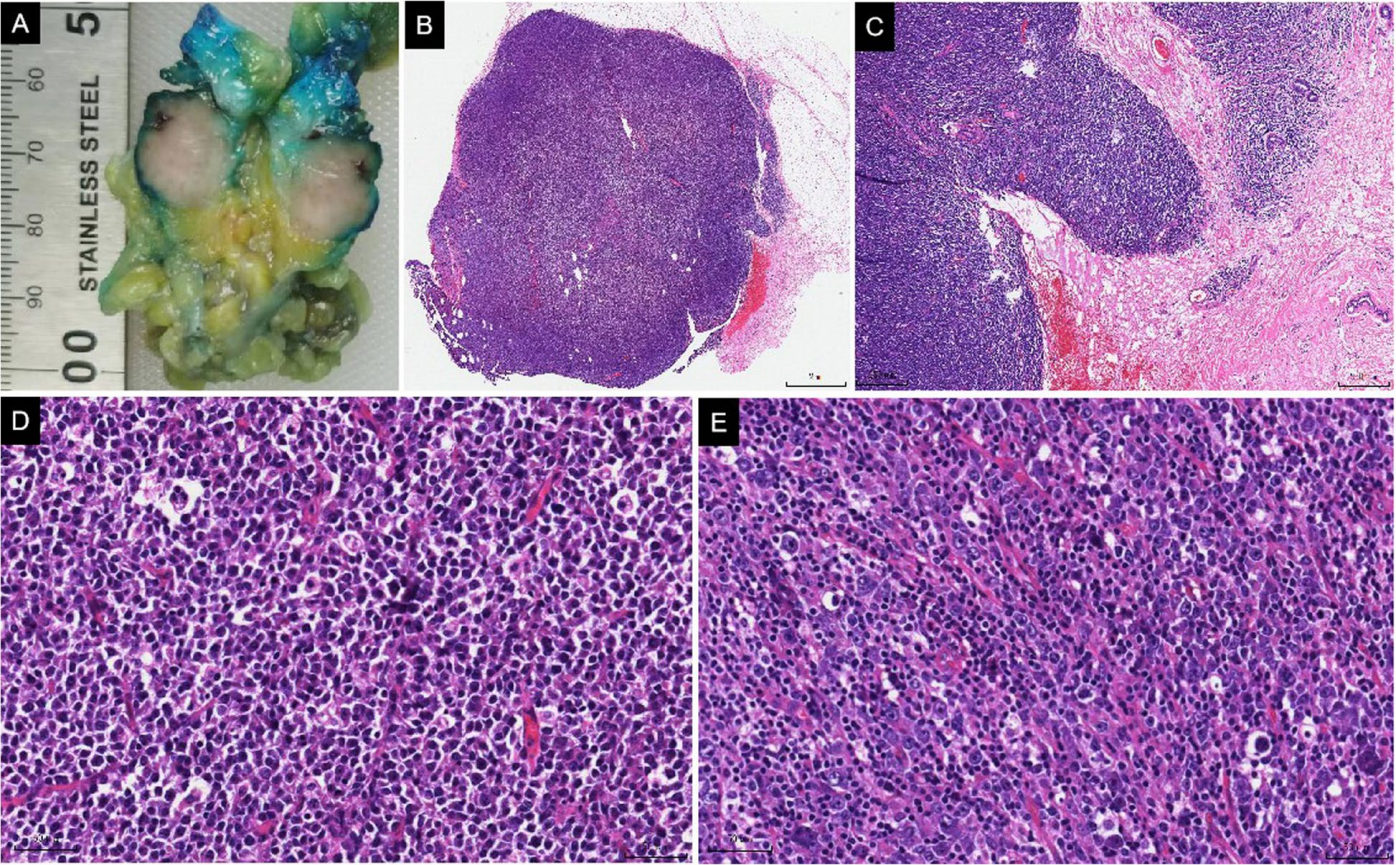
Gross examination and histomorphology features. Gross findings **A** greyish-white mass was observed in the breast tissue section, with a greyish-white medium texture and clear borders (**A**). Histological staining showed that the boundary of the mass was cleared (**B**), and small breast ducts were visible in the periphery (**C**, × 100). High magnification showed poor adhesion of tumor cells, a medium amount of cytoplasm, a high nucleoplasm ratio, obvious cell heterogeneity, deep nuclear staining (**D**, × 200), some cells with obvious nucleoli and visible nuclear fission images (2E, × 200)

Based on the clinical imaging findings and puncture pathology from local hospitals, a right breast-conserving mastectomy and sentinel lymph node biopsy were performed on November 16, 2021. Gross findings revealed a greyish-white mass with a size of 2 cm by 1.5 cm in the breast tissue section, with a greyish-white medium texture and clear borders (Fig. 2A).

**Fig. 3:**
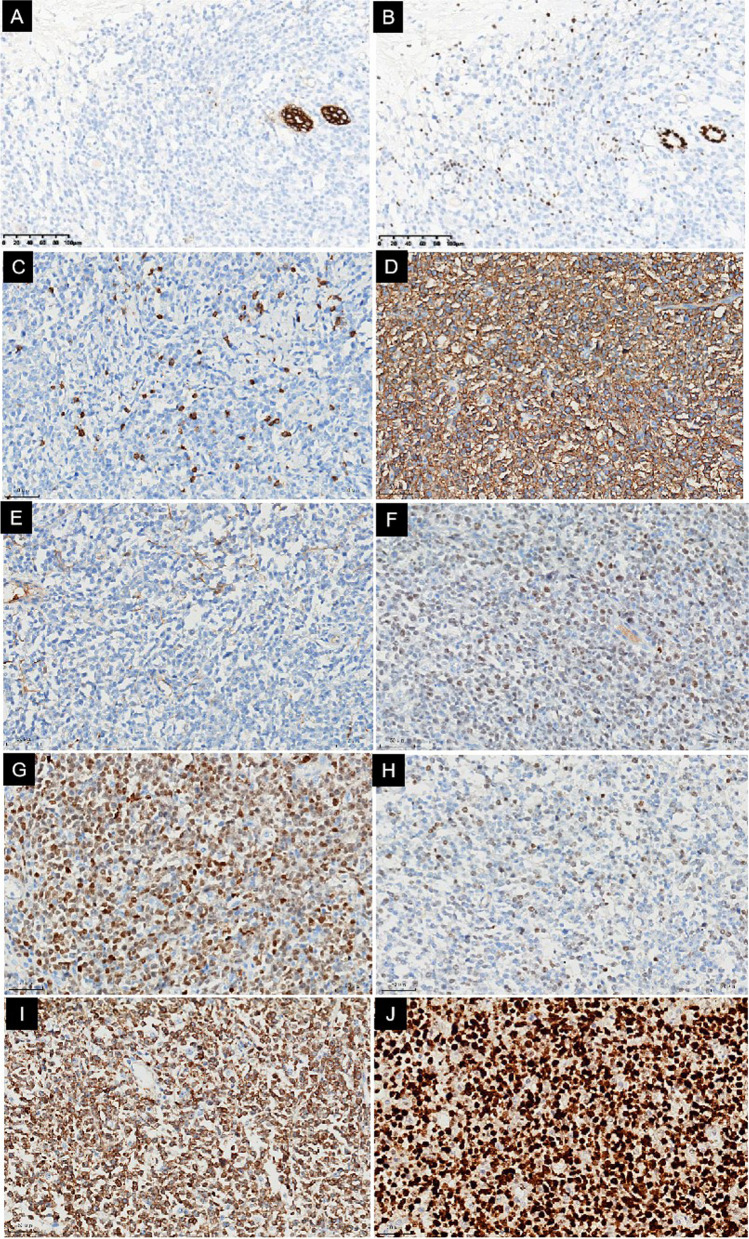
Features of immunohistochemical staining. Immunohistochemical staining showed that the tumor cells were negative for CK (AE1/AE3) and GATA3, which highlights the trapped ductal epithelium of the breast (**A**,** B**). The tumor cells were negative for CD3, which highlights T cells in the background (**C**), while the tumor cells were positive for CD20 (**D**), Bcl-6 (**F**), and MUM-1 (**G**). The tumor cells were negative for CD10 (**E**). C-Myc (Y69) (40%) (**H**), Bcl-2 (80%) (**I**), and the Ki-67 (**J**) proliferation index were high (80%). EnVision method

Histological staining showed that the boundary of the mass was cleared, and small breast ducts were visible in the periphery (Fig. 2B); tumor cells grew diffusely, and stainable vesicles were visible (Fig. 2C); high magnification showed poor adhesion of tumor cells, a medium amount of cytoplasm, a high nucleoplasm ratio, obvious cell heterogeneity, deep nuclear staining (Fig. 2D), and some cells with obvious nucleoli and visible nuclear fission images (Fig. 2E).

mmunohistochemical staining showed that the tumor cells were positive for CD20 (Fig. 3D), CD79a, PAX-5, Bcl6 (Fig. 3F), MUM-1 (Fig. 3G), CD19, c-Myc (Y69) (40%) (Fig. 3H), and Bcl2 (80%) (Fig. 3I). In addition, the tumor cells were partly positive for CD5.

The Ki-67 (Fig. 3J) proliferation index was high (80%). However, the tumor cells were negative for CD3, highlighting T cells in the background (Fig. 3C), and were negative for CK (AE1/AE3) and GATA3, highlighting the trapped ductal epithelium of the breast (Fig. 3A, B). The tumor cells were negative for estrogen receptor (ER), progesterone receptor (PR), E-cadherin, P120, CD10 (Fig. 3E), CD21, CD23, Cyclin D1, P53, ALK, and TdT. The patient underwent a bone marrow histopathologic examination on December 9, 2021, and the results showed that bone marrow hyperplasia was normal, and the ratio and distribution of granulocytes and red blood cells were not significantly abnormal. The main components of granulocytes were myelocytes and metagranulocytes. The megakaryocytes were scattered, and special staining was normal. Finally, the patient was diagnosed with PB-DLBCL with a clinical stage of IEA, and the international prognostic index (IPI) score was 1.

### Molecular pathological examination

To study the molecular features of PB-DLBCL in this male patient, we utilized an Illumina HiSeq/MiSeqDx/NextSeq assay to detect the DNA of lymphoma-related genes in paraffin-embedded tissue sample. Illumina high-throughput sequencing completely covered approximately 391,452 sites of exons, fusion-related intron regions, and variable shear regions of 121 genes associated with B cell lymphoma (Supplementary Table S 1). Based on the authoritative The Cancer Genome Atlas database, National Comprehensive Cancer Network guidelines, and 2016 World Health Organization (WHO) consensus, this detection range comprehensively covers hotspot mutations, including point mutations, small fragment insertion, and deletion mutations, gene fusion and copy number variation in the diagnostic classification of B cell lymphoma. The test results showed that the tumor-specific mutations in this sample were as follows: BCL6, TNFAIP3, PRDM1, CREBBP, DTX1, and FOXO1 (Table 1). No germline mutation was found in this sample.

**Table 1:** Tumor-specific mutation in this primary breast diffuse large B cell lymphoma in male

Tumor-specific mutations^a^ Gene	Variation	Mutant type	Mutation abundance^b^
BCL6	IGH ~ BCL6 fusion	IGH ~ BCL6: exon2	37.00%
TNFAIP3	p.E452* Exon 7 nonsense mutation	c,1354G > T(p.E452*)	67.40%
PRDM1	p.G100Efs*11 the 3rd exon frameshift mutation	C.299_324del(p.G100Efs*11)	8%
CREBBP	p.M1798I the 31st exon missense mutation	c,5394G > T(p.M1798I)	40.10%
DTX1	p.N13S the 1st exon missense mutation	c.38A > G(p.N13S)	41.80%
DTX1	p.G16A the 1st exon missense mutation,	c.47G > C(p.G16A)	41.20%
FOXO1	p.Y196C the 1st exon missense mutation	c.587A > G(p.Y196C)	10.10%

^a^Tumor-specific mutations are defined as those contained in tumor cells that are not germline mutations and are not heritable

^b^Mutation abundance is defined as the specific proportion of mutant alleles in all alleles at the site

In addition, the sample was negative for Epstein-Barr virus-encoded small RNA (EBER) by in situ hybridization (ISH). Given the dual expression of MYC and BCL2, the sample was tested for rearrangement of MYC, BCL2, and BCL6 by fluorescent in situ hybridization (FISH) using break-apart dual-color probes. The tumor cells were negative for MYC and BCL2 translocation. BCL6 translocation was detected in this sample. Moreover, MYD88 and CD79b gene mutations were wild-type by Sanger sequencing in this sample.

### Postoperative treatment and follow-up

**Fig. 4:**
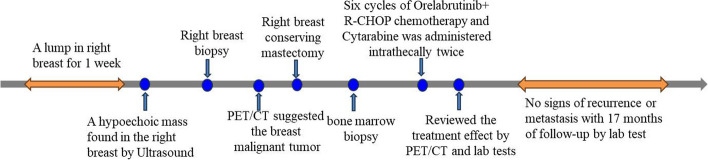
The timeline of diagnosis and treatment

After six cycles of orelabrutinib plus R-CHOP chemotherapy, cytarabine was administered intrathecally twice on April 13, 2022. In addition to routine nursing, the patient received professional psychological counseling, which improved the patient's recognition of the disease and actively cooperated with the treatment and follow-up. A re-examination of PET-CT showed no recurrence or metastasis in this patient. As of the last follow-up on Apr 23, 2023, the patient had good disease control and no recurrence. The timeline of diagnosis and treatment is shown in Fig. 4.

## Discussion

PBL is rare, accounting for 1.7–3% of all extranodal lymphomas and 0.38–0.7% of non-Hodgkin's lymphomas [24]. Most cases occur in women, with few cases reported in men. In male cases, the age range of onset is 9–85 years, with a predilection for 60 years. The onset is usually unilateral, but bilateral breast onset has also been reported [15].

Reports in the literature show that PB-DLBCL is the most common, followed by follicular lymphoma and marginal zone lymphoma. Since there were 2 studies with 6 cases of male primary breast lymphoma that did not give clinical information [1, 2], we listed 21 cases reported in the literature, including age of onset, clinical manifestations, location, mass size, sample type, pathological diagnosis, treatment, and prognosis (Table 2).

**Table 2:** Clinical features of published cases of primary breast lymphoma in males

Case	Age(year)	Clinical manifestation	Location	Size (cm)	Sample type	Diagnosis	treatment	follow-up	References
1	76	A history of bilateral gynecomastia	Lower outer quadrant, right breast	7 × 6	mastectomy	DLBCL	CT	39 months, no recurrence	[3]
2	69	A soft, mobile, and clearly defined mass	Beneath the left nipple	2.4 × 3.9	mastectomy	DLBCL	CT	12 months, no recurrence	[4]
3	67	A palpable mass	Right breast	6 × 5	mastectomy	DLBCL	CT	12 months; died	[5]
4	64	A painless, gradually growing lump	Medial area, left breast	7 × 6	CNB	DLBCL	CT + RT	12 months, CR	[6]
5	62	A mass	Left breast	7 × 4	mastectomy	MZL	CT + RT	NA	[7]
6	48	A unilateral palpable well-circumscribed mass	Left breast	3.8 × 3.5	FNA	DLBCL	CT	7 months, reduction	[8]
7	51	A palpable mass for 3-month	Upper outer quadrant, left breast	2.3	mastectomy	ALCL	CT	12 months, no recurrence	[9]
8	50	Left gynecomastia	Behind the left nipple	2 × 2	CNB	DLBCL	CT	CR	[10]
9	46	A painless mass	Right chest wall	1.6	mastectomy	FL	NA	40 months; CR	[11]
10	75	A mass for 1 month	Under the left nipple	5	CNB	DLBCL	CT + RT	8 months; no recurrence	[12]
11	56	A visible tumor	Left breast	NA	CNB	DLBCL	CT + RT	17 months; CR	[13]
12	74	A mass	Right breast	NA	CNB	DLBCL	CT	CR	[14]
13	82	Bilateral painless masses	Bilateral breast	8 × 6	CNB	HGB	CT	CR	[15]
14	81	A painless tumor	Under the left nipple	5.5 × 5.0	CNB	DLBCL	CT + RT	CR	[16]
15	72	A progressive mass persisting over 3 months	Left breast	NA	NA	HGB	NA	NA	[17]
16	74	A mass	Right breast	NA	CNB	DLBCL	CT	CR	[18]
17	64	A gradually growing lump for 2 weeks	Upper outer quadrant, left breast	2.0 × 1.9	CNB	FL	CT + RT	CR	[19]
18	65	A gradually-increasing lump	The right chest	NA	Mastectomy	DLBCL	CT	5 years, CR	[20]
19	9	A mass for 2 months ago	Under the right areola	3.5 × 1.7	Mastectomy	B-LBL	NA	NA	[21]
20	64	A painful, enlarging lump for 1 month	Left breast lump	3.8	FNA	FL	NA	NA	[22]
21	63	A painless for 3 weeks	Right retro-areolar	5.4	CNB	DLBCL	CT	NA	[23]

*CNB* Core needle biopsy, *FNA* Fine needle aspiration cytology, *NA* No available, *HGB* High-grade B cell lymphoma, *CT* Chemotherapy, *RT* Radiotherapy, *MZL* Marginal zone lymphoma, *ALCL* Anaplastic large-cell lymphoma, *FL* Follicular lymphoma, *B-LBL* B cell lymphoblastic lymphoma, *CR* Complete remission

According to the first description in 1972 by Wiseman and Liao [25], the clinical and histologic criteria for the diagnosis of PBL are as follows: (1) a close association between breast tissue and infiltrating lymphoma; (2) no evidence of widespread lymphoma and no history of previous extramammary lymphoma; and (3) documentation of the breast as the principal organ involved and the primary site. which was later modified by Hugh et al. [26], defining PBL as the infiltration of breast tissue by lymphoma with or without regional lymph nodes in patients without a history of prior nodal or extranodal lymphoma and systemic disease at the time of diagnosis.

The key diagnostic feature of pathology is that the tumor cells grow diffusely and are closely associated with the surrounding breast tissue. The tumor morphology is similar to that of DLBCL in other sites.

The histomorphology of this case should be differentiated from nonspecific types of breast cancer with medullary carcinoma features and pleomorphic lobule carcinoma. In particular, when pathological puncture biopsy samples have relatively few tumor cells and diffuse distribution and are negative for ER, PR, and HER-2, they are more likely to be confused with triple-negative breast cancer. However, on the surgical pathological specimens, it can be seen that the boundary of the mass is relatively clear, with no in situ cancer component, but more apoptosis can be seen in PB-DLBCL. Immunohistochemical results showed that the tumor cells were negative for CK (AE1/AE3), which was positive for residual breast duct epithelium, and GATA3 was negative for tumor cells and positive for residual breast duct epithelium. The tumor cells expressed the B cell markers CD20, CD79a, and PAX-5 but were negative for CD3, ER, and PR. The tumor cells were negative for CD10 but positive for BCL6 and MUM-1. According to Han's classification [27], this case was classified as non-GCB type. The tumor cells were positive for c-Myc (Y69) and Bcl2, and the Ki-67 proliferation index was up to 80%. The patient’s preoperative systemic PET/CT image showed only abnormal foci of increased glucose metabolism in the right breast and no previous history of lymphoma. Therefore, this case was consistent with PB-DLBCL, non-GBC type.

Large B cell lymphoma with an immune sanctuary is a new concept introduced by WHO-HAEM5 that encompasses aggressive B cell lymphomas of the central nervous system (CNS) of origin, the vitreoretinal compartment, and the testis of immunocompetent patients. They arise from immune refuges formed by their respective anatomical structures (such as the blood–brain barrier, blood-retinal barrier, and blood-testis barrier), as well as from immunoregulatory systems within their respective primary sites, and share a common immunophenotype and molecular profile. Patients often show an activated B cell immunophenotype (usually negative for CD10 and positive for MUM1 and BCL6) and are negative for EBER. Mutations are characterized by comutations in MYD88 and CD79B, with MHC class I and II and B2M (β2-microglobulin) gene inactivation immune escape. Some lymphomas occurring in the breast and skin can also have these features, so this group of “immune sanctuary lymphomas” may be expanded in future classifications [28]. Our case also had an activated B cell immunophenotype. In the literature, MYD88 and CD79B mutations have been reported in 27/46 (58.7%) and 11/33 (33.3%) primary breast diffuse large B cell lymphomas in females [29]. However, our case was negative for these two mutations and did not belong to the MYD88/CD79B-mutated (MCD) genotype.

Shaymaa Elgaafary et al. [17] reported a case of male breast high-grade B cell lymphoma with MYC and BCL2 rearrangement and Burkitt morphology (so-called double-hit lymphoma). Since our case had no rearrangement of MYC or BCL2, it was not consistent with “double-hit” lymphoma.

Franco F et al. [30] analyzed the mutational profile of primary lymphoma of the breast through targeted massive sequencing with a panel of 38 genes in a group of 17 female patients with primary breast diffuse large B cell lymphoma. The genes with a higher mutational frequency included PIM1 (in 50% of the analyzed samples); MYD88 (39%); CD79B, PRDM1, and CARD11 (17%); and KMT2D, TNFIAP3, and CREBBP (11%). TNFIAP3, PRDM1, and CREBBP gene mutations were found in our case. In addition, we also found DTX1 and FOXO1 mutations in this case. Wenqi Zhang et al. [31] sequenced 16 female cases by a target panel of 112 genes related to lymphoma. Next-generation sequencing (NGS) identified 203 mutations spanning 35 genes and revealed that the top high-frequency mutant genes included PIM1 (68.75%), MYD88 (56.25%), DTX1 (31.25%), CD79B (31.25%), KMT2D (31.25%), TNFAIP3 (25%), and ITPKB (25%), indicating crucial roles in lymphomagenesis and possibly consistent with the trend for shorter survival and poor prognosis. TNFAIP3 and DTX1 mutations were also detected in our sample.

An IGH/BCL6 fusion was detected in our case, which consisted of a rearrangement of the IGH gene and BCL6 gene exon 2. BCL6 belongs to the Krupple family of zinc finger proteins and encodes a transcription factor that is involved in the formation of DLBCL by acting on other proteins to inhibit gene transcription. BCL6 gene rearrangement is the most common rearrangement in DLBCL [32]. Studies have shown that DLBCL patients with BCL6 rearrangement have poor OS [33]. However, a trend toward inferior overall survival was observed in association with BCL6 rearrangement among patients treated with R-CHOP but not among patients treated with CHOP. The introduction of rituximab may have altered the prognostic impact of BCL6 gene rearrangement in patients with DLBCL [34].

A nonsense mutation in exon 7 of TNFAIP3 gene p. E452 was detected in this sample. Tumor necrosis factor alpha-induced protein 3 (TNFAIP3), a negative regulator of the NF-κB pathway, is a tumor suppressor gene in a variety of B cell lymphomas. In a study of 134 DLBCL patients, A20 deletion was found in 23.1% (31/134) of patients and 50% (14/28) in non-GBC DLBCL compared to 22.2% (4/18) in GBC DLBCL. In the rituximab treatment group, patients with A20 deletions had a relatively good prognosis (*p* = 0.0454) [35]. The TNFAIP3 gene mutation contains 3 frameshift mutations, 3 nonsense mutations, 1 missense mutation, and 1 variable shear mutation in 4 patients, which is only associated with the pathological type of PBL (*P* = 0:006). The median OS of TNFAIP3 mutant and nonmutant patients was 21 and 23 months, respectively [31].

This sample harbored a missense mutation in exon 31 of the CREBBP gene, p.M1798I. The CREBBP gene encodes histone acetyltransferase, which regulates protein and nonhistone activity with EP300. Approximately 39% of DLBCL and 41% of follicular lymphoma patients contain inactivated mutations in the CREBBP or EP300 genes and are commonly seen in GBC-DLBCL. CREBBP and EP300 mutations are one of the main pathogenic mechanisms of B cell non-Hodgkin lymphoma (B-NHL) and have a direct impact on the use of drugs targeting acetylation/deacetylation mechanisms [36]. CREBBP inactivation mutations can participate in the occurrence and development of B-NHL by reducing the acetylation of p53 and BCL6, causing the inactivation of p53 and upregulating the expression of BCL6 [37]. In addition, DLBCL patients with CREBBP or EP300 mutations had significantly poorer OS and PFS [38].

In this sample, missense mutation was detected in exon 1 of DTX1 gene p.N13S and p.G16A. The DTX1 gene encodes E3 ubiquitination ligase and a regulator of the NOTCH pathway. The frequency of DTX1 mutation in DLBCL was 14%. Compared with wild-type DTX1 patients, patients with non-synonymous DTX1 mutation had significantly reduced survival. If the mutation was located in the DTX1 WWE1 domain, TTP, PFS, and OS were reduced [39]. One study showed that the frequency of DTX1mutations in Chinese patients was 13% (16/136), all occurring in exon 1, which encodes most of the N-terminal protein interaction domains (WWE1) [40].

Missense mutation was detected in exon 1 of FOXO1 gene p.Y196C. FOXO1 is often used as a transcription factor, regulating genes involved in cell differentiation, apoptosis, and DNA damage repair. The mutation rate of FOXO1 in DLBCL was 8.6% (24/279), and most of its mutations were located in exon 1. In addition, DLBCL patients with FOXO1 mutations have a poorer prognosis [41].

Exon 3 frameshift mutation of PRDM1 gene p.G100Efs11, which may be involved in tumor development. PRDM1, also known as BLIMP-11, is a tumor suppressor gene that can inhibit the expression of a series of genes related to the proliferation and characterization of B cells and promote their plasma cell differentiation. The incidence of PRDM1 inactivated mutations in activated B cell (ABC) type DLBCL was 25%. It was found that ABC-type DLBCL patients with PRDM1 mutation had significantly lower OS and PFS after R-CHOP treatment than GCB DLBCL. PRDM1 may be a unfavorable factor in the poor prognosis of ABC-type DLBCL patients receiving R-CHOP [42].

Due to the low incidence of PB-DLBCL, the treatment strategy lacks high-level evidence-based medicine. At present, extensive radical mastectomy is not advocated. Surgery only needs to meet the pathological diagnosis, and mastectomy will delay the treatment time. At present, PB-DLBCL is treated with anthracycline-based chemotherapy regimens, such as CHOP regimen [2]. Most studies have shown that rituximab can prolong the survival of PB-DLBCL patients and effectively reduce the CNS recurrence rate [2, 43]. Radiotherapy can consolidate the effect of systemic chemotherapy, and radiotherapy combined with chemotherapy can reduce the local recurrence of breast cancer [44].

Just as Golia D'Augè T et al. have shown reductions in morbidity and mortality, improved patient outcomes, reduced disease management costs, and extended follow-up in gynecological cancers [45], such strategies should be maintained in breast lymphoma. Genetic mutations are often observed in patients with breast lymphoma. Hospital staff and patients should pay attention to genetic mutations that are adverse to the course of the disease. Patients should be reviewed every 3 months for 1 year to monitor for recurrence. When the patient does not review on time, the hospital staff should remind the patient to go to the hospital for review.

In conclusion, we describe the male patient with PB-DLBCL, the non-GBC type, dual-expression type, with IgH/BCL6 fusion, nonsense mutation in TNFAIP3, frameshift mutations in PRDM1, missense mutations in CREBBP, DTX1, and FOXO1. The present case adds to the understanding of the gene mutation profiles of PB-DLBCL in males.

### Supplementary Information

**Additional file 1: Supplementary Table S1.** List of 121 genes related to B-cell lymphoma.

## Data Availability

The original contributions presented in the study are included in the article/Supplementary Material. Further inquiries can be directed to the corresponding author.
