# Correction: Clinicopathologic and mutational profiles of primary breast diffuse large B cell lymphoma in a male patient: case report and literature review

**DOI:** 10.1186/s12957-023-03259-4

**Published:** 2023-12-12

**Authors:** Fengbo Huang, Yachao Ruan, Xiaojuan He, Hui Lian, Jinhua Yang

**Affiliations:** 1 Department of Pathology, The Second Affiliated Hospital of Zhejiang University School of Medicine, Hangzhou, China; 2 Key Laboratory of Tumor Microenvironment and Immune Therapy of Zhejiang Province, Hangzhou, China; 3 Department of Radiology, The First Affiliated Hospital of Zhejiang University School of Medicine, Hangzhou, China; 4 Linping Campus, The Second Affiliated Hospital of Zhejiang University School of Medicine, Hangzhou, China; 5 Department of Hematology, The First Affiliated Hospital of Zhejiang Chinese Medical University (Zhejiang Provincial Hospital of Chinese Medicine), Hangzhou, China

**Correction: World J Surg Onc 21, 342 (2023)**

**https://doi.org/10.1186/s12957-023-03234-z**

Following publication of the original article [1], the author reported that affiliation 5 should be corrected from

Department of Hematology, The First Affiliated Hospital of Zhejiang Traditional Chinese Medicine University, Hangzhou, China

To

Department of Hematology, The First Affiliated Hospital of Zhejiang Chinese Medical University (Zhejiang Provincial Hospital of Chinese Medicine, Hangzhou, China

The original article has been corrected.
