# Supplementary material for: Clinicopathologic and mutational profiles of primary breast diffuse large B cell lymphoma in a male patient: case report and literature review
Source: World J Surg Oncol. 2023 Oct 26;21:342. doi: 10.1186/s12957-023-03234-z (PMC10601260; doi:10.1186/s12957-023-03234-z)
Supplement: Supplementary file 1 — Additional file 1: Supplementary Table S1. List of 121 genes related to B-cell lymphoma. [file 12957_2023_3234_MOESM1_ESM.docx]

Supplementary table 1：list of 121 genes related to B-cell lymphoma

| ALK | | CDKN2B | | GNA13 | | MYC |  | SGK1 | |
| --- | --- | --- | --- | --- | --- | --- | --- | --- | --- |
| AKT1 | | CDKN2C | | HRAS | | MYD88 |  | SF3B1 | |
| AKT2 | | CD274 | | ID3 | | NF1 | | SOCS1 | |
| APC | | CD70 | | IKZF1 | | NFKBIA | | SPEN | |
| ARID1A | | CD79A | | INPP5D | | NFKB1 | | SMARCA4 | |
| ARID1B | | CD79B | | IRF4 |  | NFKB2 | | STAT3 | |
| ARID2 | | CD83 | | JAK1 | | NFKBIE | | STAT6 | |
| ATM | | CD58 | | JAK2 | | NOTCH1 | | SYK | |
| ATR | | CIITA | | JAK3 | | NOTCH2 | | TBL1XR1 | |
| B2M | | CREBBP |  | KDM6A | | NRAS | | TCF3 | |
| BCL10 | | CTLA4 | | KDR | | PAX5 | | TET2 | |
| BCL2 |  | CTNNB1 | | KIT | | PDCD1LG2 | | TNFAIP3 | |
| BCL6 |  | CXCR4 | | KLHL6 | | PIK3R1 | | TNFRSF14 | |
| BCOR | | CYLD | | KMT2A | | PIK3CA | | TP53 |  |
| BCORL1 | | DDX3X | | KMT2C | | PIK3CD | | TP63 | |
| BIRC3 | | DNMT3A | | KMT2D | | PIM1 | | TP73 | |
| BTK | | DTX1 | | KRAS | | PLCG2 | | TRAF2 | |
| BRAF | | EP300 |  | LYN | | POT1 | | TRAF3 | |
| CALR | | ERBB4 | | MALT1 | | PRDM1 | | TRAF5 | |
| CARD11 | | E2H2 |  | MAP2K1 | | PTEN | | WHSC1 | |
| CCND1 | | FAS | | MAP3K14 | | PTPN6 | | XPO1 | |
| CCND2 | | FAT1 | | MDM2 | | PTPRD | |  |  |
| CCND3 | | FBXO11 | | MED12 | | RB1 | |  |  |
| CDKN1B | | FBXW7 | | MEF2B | | ROS1 | |  |  |
| CDKN2A | | FOXO1 | | MTOR | | SETD2 | |  |  |
|  |  |  |  |  |  |  |  |  |  |
|  |  | Diagnostic key gene | |  |  |  |  |  |  |
|  |  | Prognostic gene | |  |  |  |  |  |  |
|  |  | target key genes for druges | | |  |  |  |  |  |
